# Estrogen Therapy Worsens Cardiac Function and Remodeling and Reverses the Effects of Exercise Training After Myocardial Infarction in Ovariectomized Female Rats

**DOI:** 10.3389/fphys.2018.01242

**Published:** 2018-09-05

**Authors:** Simone Alves de Almeida, Erick R. G. Claudio, Vinicius Mengal, Girlandia A. Brasil, Eduardo Merlo, Priscila L. Podratz, Jones B. Graceli, Sonia A. Gouvea, Gláucia Rodrigues de Abreu

**Affiliations:** ^1^Laboratório de Regulação Neurohumoral da Circulação, Departamento de Ciências Fisiológicas, Centro de Ciências da Saúde, Universidade Federal do Espírito Santo, Vitória, Brazil; ^2^Núcleo de Pesquisas em Ciências Farmacêuticas – Nupecfarma, Programa de Pós-graduação em Ciências Farmacêuticas, Universidade Vila Velha, Vila Velha, Brazil; ^3^Departamento de Morfologia, Centro de Ciências da Saúde, Universidade Federal do Espírito Santo, Vitória, Brazil

**Keywords:** ovariectomy, estrogen therapy, cardiac function, remodeling, oxidative stress

## Abstract

There is an increase in the incidence of cardiovascular events such as myocardial infarction (MI) after menopause. However, the use of estrogen therapy (E2) remains controversial. The aim of this study was to evaluate the effects of E2, alone and combined with exercise training (ET), on cardiac function and remodeling in ovariectomized (OVX) rats after MI. *Wistar* female rats underwent ovariectomy, followed by MI induction were separated into five groups: S; MI; MI+ET; MI+E2; and MI+ET+E2. Fifteen days after MI or sham surgery, treadmill ET and/or estrogen therapy [17-β estradiol-3-benzoate (E2), *s.c.* three times/week] were initiated and maintained for 8 weeks. After the treatment and/or training period, the animals underwent cardiac hemodynamic evaluation through catheterization of the left ventricle (LV); the LV systolic and diastolic pressures (LVSP and LVEDP, respectively), maximum LV contraction and relaxation derivatives (dP/dt+ and dP/dt−), and isovolumic relaxation time (Tau) were assessed. Moreover, histological analyses of the heart (collagen and hypertrophy), cardiac oxidative stress [advanced oxidation protein products (AOPPs)], pro- and antioxidant protein expression by Western blotting and antioxidant enzyme activity in the heart were evaluated. The MI reduced the LVSP, dP/dt+ and dP/dt− but increased the LVEDP and Tau. E2 did not prevent the MI-induced changes in cardiac function, even when combined with ET. An increase in the dP/dt+ was observed in the E2 group compared with the MI group. There were no changes in collagen deposition and myocyte hypertrophy caused by the treatments. The increases in AOPP, gp91-phox, and angiotensin II type 1 receptor expression induced by MI were not reduced by E2. There were no changes in the expression of catalase caused by MI or by the treatments, although, a reduction in superoxide dismutase (SOD) expression occurred in the groups subjected to E2 treatment. Whereas there were post-MI reductions in activities of SOD and catalase enzymes, only that of SOD was prevented by ET. Therefore, we conclude that E2 therapy does not prevent the MI-induced changes in cardiac function and worsens parameters related to cardiac remodeling. Moreover, E2 reverses the positive effects of ET when used in combination, in OVX infarcted female rats.

## Introduction

Myocardial infarction (MI) is considered a highly prevalent disease in the western countries (Mozaffarian et al., 2016); it results from the interruption of blood flow in the coronary arteries, which causes hypoxia and tissue injury (Sukumalchantra et al., 1970). The causes of MI are diverse, and both environmental factors and hormonal factors can influence its development.

The prevalence of MI is lower in women than in men (Mendis et al., 2011). However, the values become equal or even inverted in women older than 60 years (Kannel, 2002). At that age, there is an abrupt drop in the production of female sex hormones, a period known as menopause (Puzianowska-Kuznicka, 2012).

Several studies indicate that there is a relationship between the drop in estrogen (E2) production by the gonads and the development of MI, suggesting a protective effect of E2 against MI in women (Van Eickels et al., 2003; Terrell et al., 2008; Piro et al., 2010; Santos et al., 2014). Nonetheless, the effects of estrogen therapy on the cardiovascular system remain controversial (Chen et al., 2009; Boardman et al., 2015). In fact, in an animal model of menopause and MI, estrogen therapy led to a reduction in myocardial wall peak tension and inhibition of dilation of the left ventricle (LV) (Smith et al., 2000). In contrast, Van Eickels et al. (2003) observed increased mortality and cardiac remodeling in ovariectomized (OVX) mice after MI.

The mechanisms involved in the cardiovascular functions of E2 are diverse. There is evidence showing the antioxidant effects of this hormone, which promotes an increase in the bioavailability of endothelial nitric oxide (NO), resulting in vasodilation and subsequent reduction in peripheral vascular resistance (Hernández et al., 2000). This vasoprotective effect was attributed to increased expression of the superoxide dismutase (SOD) enzyme, which is responsible for the degradation of the superoxide anion (Borrás et al., 2010; Campos et al., 2014), the main free radical produced in vessels and the one responsible for NO depletion (Judkins et al., 2010). Moreover, E2 influences the humoral pathways of cardiovascular control, such as the renin-angiotensin-aldosterone system (RAAS) (Shenoy et al., 2009). Nickenig et al. (2000) demonstrated that estrogen is capable of promoting a reduction in the gene expression and half-life of the angiotensin II type 1 (AT-1) receptor in vascular smooth muscle cells, indicating a modulating effect of E2 on this system.

In addition to endogenous effects, environmental factors are important in the development of cardiovascular diseases (CVDs). The practice of regular physical exercise has been suggested to be of great importance in the prevention and treatment of CVD, including for post-menopausal women (Moilanen et al., 2012). The benefits of exercise training (ET) in the post-menopausal period, described in clinical studies and in experimental models of ovariectomy, are many (Moilanen et al., 2012; Endlich et al., 2013; Grindler and Santoro, 2015; Mazurek et al., 2017). In a systematic review, Grindler and Santoro (2015) showed that ET promotes a significant improvement in several cardiovascular co-morbidities such as hypertension, coronary disease, and infarction, among others, in addition to metabolic factors such as dyslipidemia, diabetes, and obesity.

ET and E2 are capable of modulating several signaling pathways, promoting improvement in several cardiovascular regulatory factors, such as modulating the expression of angiotensin II receptors (AT-1 and AT2), which are involved in the pathophysiology of several CVDs including MI (Adams et al., 2005).

It is known that the RAAS is closely related to oxidative stress and that the activation of this system promotes the formation of reactive oxygen species (ROS), mainly via NAD(P)H oxidase (Virdis et al., 2011). This enzyme is the main responsible for the formation of superoxide anions in the heart tissue via its gp91-phox subunit (Fukui et al., 2001). Additionally, we have previously shown that the expression of this enzyme is elevated in the post-infarction tissue resulting in increased cardiac oxidative stress (de Almeida et al., 2014).

Therefore, we observe that E2 and ET modulate important signaling pathways that may help in the prevention of heart damage caused by MI. However, little is known about the effects of post-MI E2 replacement on the heart as well as whether its combination with ET can promote additive effects. Taking this into consideration, the objective of this study was to evaluate the effects of treatment with E2, alone and in combination with ET, on cardiac function and remodeling in estrogen-deficient female rats after MI. Moreover, we analyzed the influence of pathways associated with the production and degradation of ROS in this process.

## Materials and Methods

### Animals

Eight-week-old female Wistar rats (*Rattus norvegicus*), weighing 250–300 g were used in this study. The animals were provided by the Health Sciences Centre facility and were housed in collective cages with free access to water and food (Purina Labina, Brazil). The room temperature (22 and 24°C) and the light/dark cycle (12 h–12 h) were controlled. The study was approved by the Animal Research Ethics Committee of the Health Sciences Centre (Protocol 059/2012), and all of the experimental procedures were conducted according to the “National Institute of Health [NHI] (2010) Guide for the Care and Use of Laboratory Animals.”

### Ovariectomy

Ovariectomy was performed in all of the experimental groups, as previously described (de Almeida et al., 2014). In summary, the animals were anesthetized with a mixture of ketamine and xylazine (50 and 10 mg/kg i.p., respectively). Following anesthesia, an incision was made in the skin 1 cm from the median line between the last costal margin and the thigh, followed by an incision in the muscular layer and exposure of the peritoneal cavity for the removal of the ovaries (ovariectomy) and ligation of the uterine tubes on both sides. After the procedure, the muscles and the skin were sutured and rinsed, and the animals received an injection of antibiotic (0.1 mL of 2.5% enrofloxacin intramuscularly, i.m.).

### Induction of Myocardial Infarction

Seven days after the ovariectomy, the animals were anesthetized with ketamine and xylazine (50 and 10 mg/kg i.p., respectively). A lateral incision at the level of the fourth intercostal space was made to expose the heart. The heart was eviscerated, and the left anterior descending coronary artery was permanently occluded with a mononylon 6.0 thread assembled on a non-traumatic needle. After the occlusion, the chest was immediately closed and sutured.

The SHAM group (S) underwent a sham surgical procedure, which consisted of the application of all the steps previously described, except for the occlusion of the coronary artery (Baldo et al., 2012). Upon MI induction, the animals were randomly divided into the following groups (*n* = 10 each): S group, sedentary MI group (MI), MI group undergoing ET (MI+ET), MI group receiving estrogen therapy (MI+E2), and MI group undergoing ET and receiving estrogen therapy (MI+ET+E2).

### Treadmill Running Protocol

The running training was conducted using an electric treadmill (EP 131, Insight, Brazil) 2 weeks after MI induction, as previously described (de Almeida et al., 2014). The training period was divided into two phases. The first was a 1-week adaptation phase (10 min/day at 0.3 km/h) with progressive increases in time until the 5th day when the maximum time of 60 min was reached. From the 2nd week on, the duration of exercise was constant (60 min/day), but the exercise intensity was gradually increased from 0.3 km/h until reaching a speed of 1.2 km/h. ET was performed five times per week for a total of 8 weeks.

### Estrogen Therapy

Two weeks after MI induction, E2 therapy was initiated with subcutaneous injections of estradiol-3-benzoate (5 μg; Sigma Chemical, St. Louis, MO, United States) diluted in 0.1 mL of corn oil (used as vehicle), three times per week, for a total of 8 weeks (Saengsirisuwan et al., 2009). The protocol for estrogen therapy in ovariectomized rats was based on previous studies from our group, which demonstrate that the dose used is capable to maintain the plasmatic physiological concentration of E2 observed in control rats (Claudio et al., 2013; Endlich et al., 2017). The groups that did not receive estrogen therapy received the same volume of vehicle (corn oil).

### Assessment of Cardiac Function

Forty-eight hours after the last training day, the animals were anesthetized with ketamine and xylazine (50 and 10 mg/kg i.p., respectively) for measurement of the blood pressure and LV function. A polyethylene catheter (PE50) with heparinised saline (50 U/mL Ariston sodium heparin, São Paulo, Brazil) was introduced into the left carotid artery, followed by connection to a pressure transducer (FE221 Bridge Amp, ADInstruments, Australia), which was coupled to a data acquisition system (Powerlab 4/35). The catheter was carefully guided to the LV, where the LV systolic pressure (LVSD) and LV end-diastolic pressure (LVEDP) were measured, as was the maximum LV contraction and relaxation derivatives (dP/dt+ and dP/dt−, respectively). The isovolumic relaxation time (Tau) was also measured.

### Tissue Collection

Following the hemodynamic evaluation, the animals, still under anesthesia, were euthanised by an anesthetic overdose, the heart was quickly removed and rinsed with saline, and the ventricles were separated from the atria and weighed. The ventricles were stored in formalin buffer for the histological analyses or at −80°C for the gene expression and oxidative stress assessments. The lungs were removed, rinsed, dried and weighed. Next, they were placed in a laboratory oven to determine the water percentage (Lutz, 2008). The uterus was removed and weighed to check the efficiency of estrogen therapy. Additionally, the initial body weight (IBW) and the final body weight (FBW) of the animals were recorded with the aim of confirming weight gain.

### Western Blotting

The LV samples were homogenized in lysis buffer containing NaCl (150 mM), Tris-HCl (50 mM), EDTA⋅2Na (5 mM), MgCl_2_ (1 mM) and protease inhibitor (Sigma Fast, Sigma Aldrich, United States).

The protein concentration was determined by the Bradford method (Bradford, 1976) using bovine serum albumin (BSA) as the standard. Equal amounts of protein (50 μg) were analyzed through electrophoresis (2:30 h, 80 V) on a 10% polyacrylamide gel (SDS-PAGE). Then, the proteins were transferred to polyvinylidene fluoride (PVDF) membranes for 1:40–2:30 h at 60 V in a humid blotting system. After the transfer, the membrane was blocked with tris-buffered saline with Tween (TBS-T) and fat-free milk (5%) for 2:30 h and the membranes were washed and incubated for 4 h with mouse monoclonal antibodies for catalase (CAT; 1:2000; Sigma, United States) as well as rabbit polyclonal antibodies for SOD (SOD-2; 1:500; Sigma, United States), gp91-phox (1:1000; BD, Franklin Lakes, NJ, United States) and AT-1 (1:500; Santa Cruz Biotechnology, Santa Cruz, CA, United States). After another washing with TBS-T, the membranes were incubated with anti-mouse IgG (1:3000, Inc. Abcam, Cambridge, MA, United States) or anti-rabbit IgG (1:7000; Santa Cruz Biotechnology, Santa Cruz, CA, United States) according to the antibodies’ specifications. The bands were visualized using the NBT/BCIP system (Invitrogen Corporation, Carlsbad, CA, United States) and quantified with ImageJ software [National Institutes of Health (NIH)]. The same membranes were used to determine the expression of β-actin using a mouse monoclonal antibody for β-actin (1:5000; Sigma, United States), and the results were calculated through the ratio of the density of the protein of interest to the density of the control protein (β-actin).

### Determination of Advanced Oxidation Protein Products

The advanced oxidation protein products (AOPPs) were determined in heart tissue by spectrophotometry. The heart tissue was homogenized and diluted (1:5) in phosphate-buffered saline (PBS; pH 7.4). Next, 200 μL of the sample was added to a 96-well plate together with 10 μL of potassium iodide (1.16 M) and 20 μL of glacial acetic acid. The reading was taken immediately at 340 nm with an ELISA reader (Filter Max F5 Multi-Mode Microplate Readers, Molecular Devices, Silicon Valley, CA, United States). The blank was prepared by replacing the sample with PBS. The AOPP concentrations are expressed as μM of chloramine-T/mg of protein (Valente et al., 2013).

### Antioxidant Enzyme Activity

The activities of the antioxidant SOD and catalase enzymes were measured using spectrophotometry. For the determination of SOD, the technique described by Misra and Fridovich (1972), based on the auto-oxidation of epinephrine, was used. For this purpose, the previously homogenized heart tissue samples were mixed with the reaction medium containing carbonate buffer (0.2 M; pH 10.2) and KCl (0.015 M). The reaction was started by adding epinephrine solution (0.025 M), and the readings were taken at 480 nm at intervals of 15 s for 1 min with a spectrophotometer (T80+ UV/VIS Spectrometer, Pg Instruments, Ltd., United Kingdom). The results are expressed as USOD/mg protein, with one unit of SOD considered the quantity capable of reducing the auto-oxidation of epinephrine by 50%.

For the measurement of the catalase activity, the method described by Aebi (1984) was used. Briefly, 40 μL of hydrogen peroxide (0.066 M) was added to the reaction medium containing 3 mL of phosphate buffer (0.050 M; pH 7.4). Subsequently, 60 μL of the sample was mixed, and the reading was taken at 240 nm, with the value recorded every 15 s for a minute (T80+ UV/VIS Spectrometer, Pg Instruments, Ltd., United Kingdom). The results are expressed as the extinction coefficient of peroxide per minute (ΔE⋅min/mg protein).

### Histological and Cardiac Hypertrophy Analysis

The tissues were kept in formalin buffer and then processed and embedded in paraffin. Transverse sections (4 μm) were obtained and stained with hematoxylin and eosin (H/E) and Picrosirius Red for the assessment of cardiac hypertrophy and the area occupied by collagen, respectively. The images were captured with a digital camera (Evolution, Media Cybernetics, Inc., Bethesda, MD, United States) coupled to an optical microscope (Eclipse 400, Nikon) with a magnification of 400×. Images for the quantification of collagen deposition and determination of the myocyte transverse section area were processed with ImageJ software (NIH, United States).

### Infarction Size Assessment

The epicardium perimeter related to the infarction area was determined using Picrosirius Red staining. All of the procedures were repeated for the endocardium. The slides stained with Picrosirius Red were digitalised (LaserJet Pro M1132, HP, United States) and analyzed with ImageJ software (NIH, United States). The infarction size was presented as the average percent value of the LV infarction perimeter (Fraccarollo et al., 2008).

### Statistical Analysis

The data are presented as the mean ± SEM. The data distribution was evaluated with the D’Agostino-Pearson test. The data of organ weights, protein expression, enzyme activity, AOPP and hemodynamic parameters were evaluated by the *One-way* Analysis of Variance (ANOVA), followed by the Fisher’s *post hoc* test. The histological analyses were evaluated by the non-parametric Kruskal–Wallis test, followed by the Dunn’s *post hoc* test. Values of alpha lower than 5% were considered statistically significant.

## Results

### Weight Data Analysis

Regarding the analysis of the weight data (**Table 1**), there were no differences in body mass between animals in the MI group when compared to the S group (*p* > 0.05) at the end of the experimental protocol, but the animals that received E2 alone or in combination with ET presented a lower FBW (*p* < 0.05 vs. MI and S).

**Table 1:** Effects of E2 and ET on the morphometric parameters.

	S	MI	MI+ET	MI+E2	MI+ET+E2
	(*n* = 10)	(*n* = 10)	(*n* = 10)	(*n* = 10)	(*n* = 10)
BW initial (g)	235.4 ± 15.1	215.7 ± 8.5	211.4 ± 4.8	211.7 ± 11.9	222.3 ± 8.8
BW final (g)	336.6 ± 5.9	332 ± 8.4	330.6 ± 11.4	287.1 ± 7.6^∗#†^	256.6 ± 6.8^∗#†§^
UW (g)	0.1212 ± 0.008	0.0922 ± 0.004	0.0927 ± 0.004	0.4894 ± 0.038^∗#†^	0.4025 ± 0.024^∗#†§^
Lung (g)	1.70 ± 0.05	2.02 ± 0.2325	2.42 ± 0.32	2.51 ± 0.25^∗^	2.67 ± 0.47^∗^
Lung/BW (mg/g)	4.95 ± 0.23	10.73 ± 1.28^∗^	6.83 ± 0.77^#^	10.8 ± 1.39^∗†^	11.54 ± 1.84^∗†^
Lung water (%)	80.08 ± 0.45	82 ± 0.66^∗^	81.81 ± 0.49	81.49 ± 0.56	82.26 ± 0.72^∗^

*Data are expressed as mean ± SEM. BW, body weight; UW, uterine weight. One-way ANOVA followed by the Fisher’s post hoc test. ^∗^p < 0.05 vs. SHAM; ^#^p < 0.05 vs. MI; ^§^p < 0.05 vs. MI+E2; ^†^p < 0.05 vs. MI+ET.*

For pulmonary congestion, there was an increase in the water content in the lungs in the MI group, as expected. This increase was maintained when E2 was combined with ET (**Table 1**). Treatment with either ET or E2 alone prevented the accumulation of water in the lungs.

The uterine weight was used to evaluate the efficacy of the hormone replacement therapy with E2. As expected, the MI+E2 and the MI+ET+E2 groups presented increased wet uterine weights compared with the S, MI, and MI+ET groups (*p* < 0.05).

### Infarction Size

**FIGURE 1:**
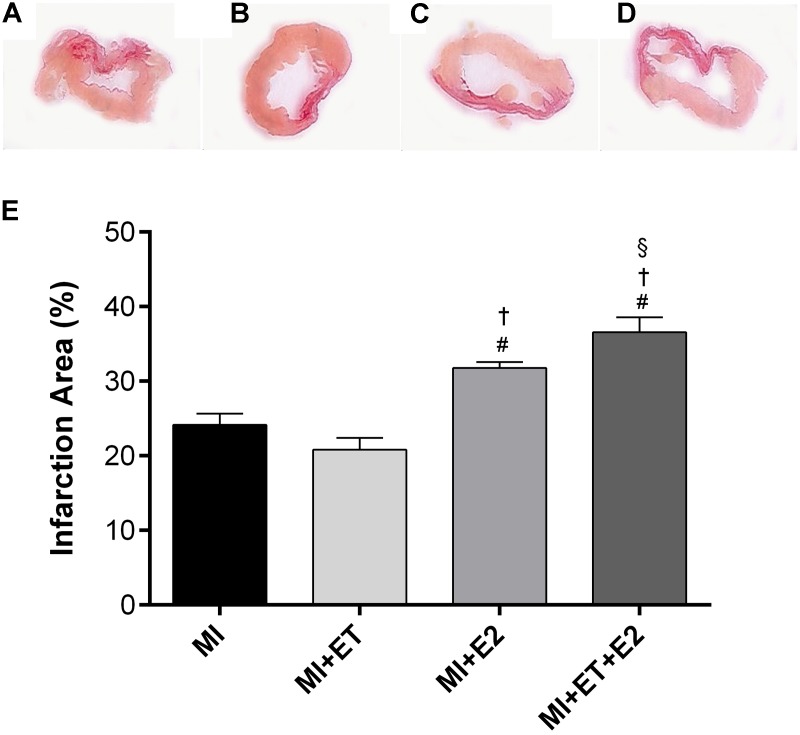
Histological analysis of myocardial infarction (MI) area. Representative images are shown in **(A–D)** and representative figure of the results are depicted in **(E)**. Data are expressed as Mean ± SEM. Kruskal–Wallis test followed by the Dunn’s *post hoc* test. ^#^*p* < 0.05 vs. to MI; ^†^*p* < 0.05 vs. to ET; ^§^ *p* < 0.05 vs. to E2.

No differences in infarction size were observed between the MI and the MI+ET groups (*p* > 0.05) (**Figure 1**). In contrast, the percentages of infarction size for the groups receiving estrogen therapy, in combination or not with ET, were significantly greater than those for the MI and MI+ET groups (*p* < 0.05).

### Cardiac Function

**FIGURE 2:**
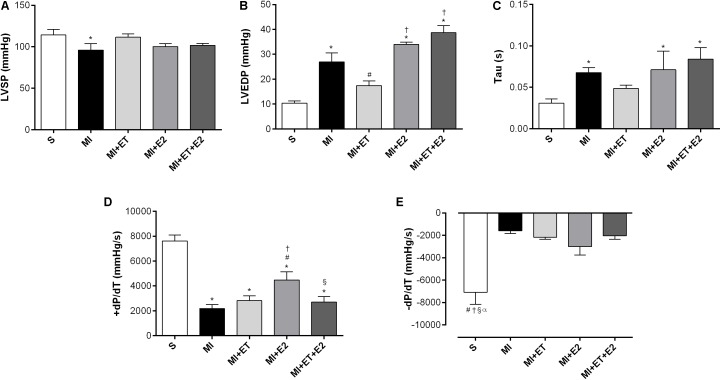
Left ventricle (LV) function measurements. **(A)** left ventricle systolic pressure (LVSP); **(B)** left ventricle end diastolic pressure (LVEDP); **(C)** isovolumetric relaxation time (Tau); **(D)** maximum rate of pressure development (+dP/dT); **(E)** maximal relaxation rate (−dP/dT). Data are expressed as Mean ± SEM. *One-way* ANOVA followed by the Fisher’s *post hoc* test. ^∗^*p* < 0.05 vs. S; ^#^*p* < 0.05 vs. MI; ^†^*p* < 0.05 vs. ET; ^§^ *p* < 0.05 vs. E2; ^α^*p* < 0.05 vs. MI+ET+E2.

In the case of cardiac functional parameters, the infarction promoted a reduction in the LVSP and an increase in the LVEDP (*p* < 0.05 vs. S) (**Figures 2A,B**). None of the interventions promoted the recovery of the LVSP, and it was also not possible to prevent the increase in the LVEDP caused by treatment with E2, in combination or not with ET. ET alone presented an opposite effect (**Figure 2B**). Additionally, there was an increase in Tau after MI (*p* < 0.05 vs. S). Treatment with E2 and its combination with ET were not able to prevent this increase. However, ET alone remained the same as for the S group (**Figure 2C**). Although treatment with E2 did not prevent the increase in Tau, treatment with E2 led to an increase in +d*P*/dt when compared to the other infarction groups. Additionally, when E2 was combined with ET, this treatment was unable to prevent this reduction (**Figure 2D**). None of the treatments modified the −dP/dt (**Figure 2E**).

### Histological Analyses

**FIGURE 3:**
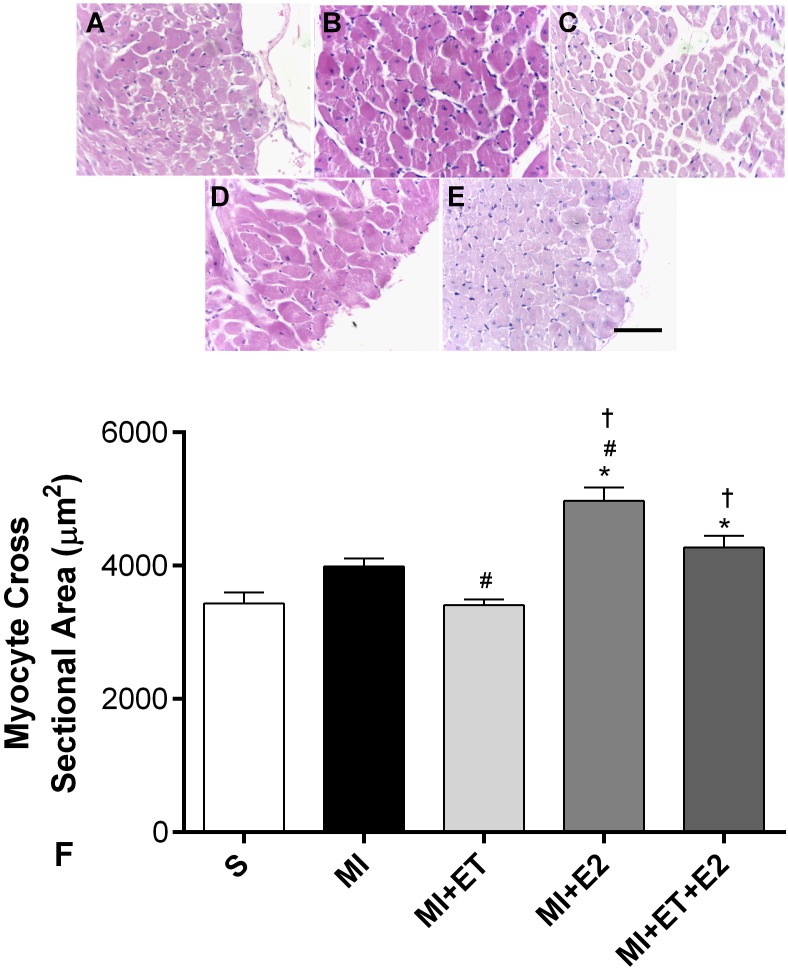
Histological analysis of myocyte cross-sectional area. Representative images are shown in **(A–E)** and representative figure of the results are depicted in **(F)**. Data are expressed as Mean ± SEM. Kruskal–Wallis test followed by the Dunn’s *post hoc* test. ^∗^*p* < 0.05 compared to S; ^#^*p* < 0.05 compared to MI; ^†^*p* < 0.05 compared to ET. Magnifier: 400×. Bar: 50 μm.

The histological analyses showed that there was an increase in cardiac hypertrophy in animals treated with E2 alone and in combination with ET, as determined by the cardiomyocytes transverse section area (**Figure 3**). This was not observed in animals undergoing ET (*p* < 0.05 vs. MI).

**FIGURE 4:**
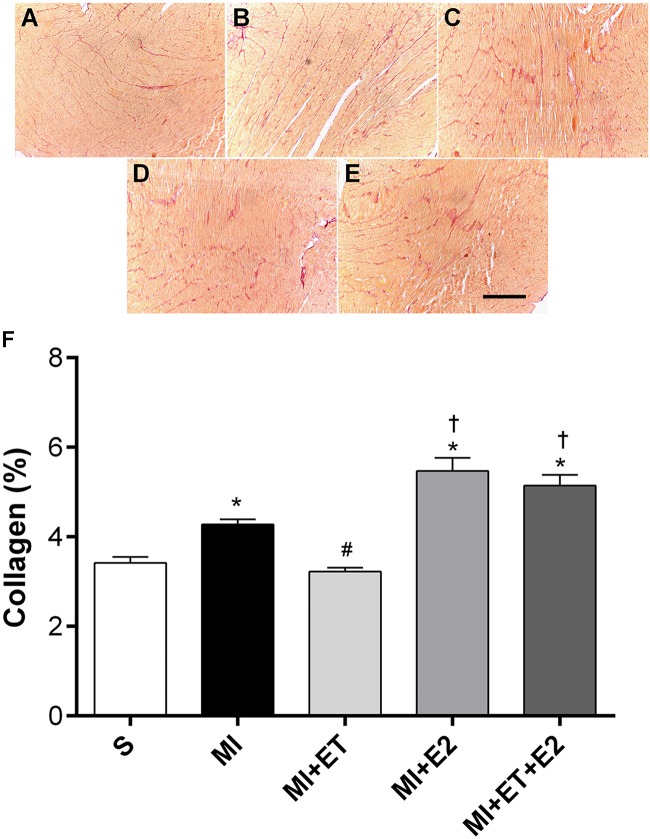
Histological analysis of interstitial collagen deposition. Representative images are shown in **(A–E)** and representative figure of the results are depicted in **(F)**. Data are expressed as Mean ± SEM. Kruskal–Wallis test followed by the Dunn’s *post hoc* test. ^∗^*p* < 0.05 compared to S; ^#^*p* < 0.05 compared to MI; ^†^*p* < 0.05 compared to ET. Magnifier: 400×. Bar: 200 μm.

As expected, MI induced an increase in the collagen deposition in the heart (*p* < 0.05), and ET was able to prevent this change (**Figure 4**). However, treatment with E2 increased the collagen deposition area, regardless of combination with ET (*p* < 0.05).

### Advanced Oxidation Protein Products

**FIGURE 5:**
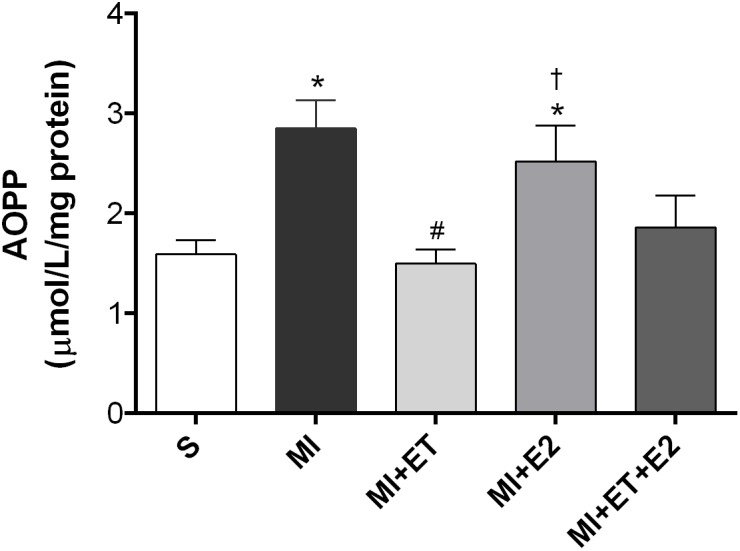
Analysis of advanced oxidation protein products (AOPPs) in the cardiac tissue. Data are expressed as Mean ± SEM. *One-way* ANOVA followed by the Fisher’s *post hoc* test. ^∗^*p* < 0.05 vs. S; ^#^*p* < 0.05 vs. MI; ^†^*p* < 0.05 vs. ET.

An increase in protein oxidation in the MI group was observed (*p* < 0.05 vs. S). Treatment with E2 was not able to prevent this increase (*p* < 0.05 vs. MI and MI+ET), not even when combined with ET (**Figure 5**). As expected, ET prevented the increase in this parameter (*p* < 0.05 vs. MI).

### Pro- and Antioxidant Protein Expression

**FIGURE 6:**
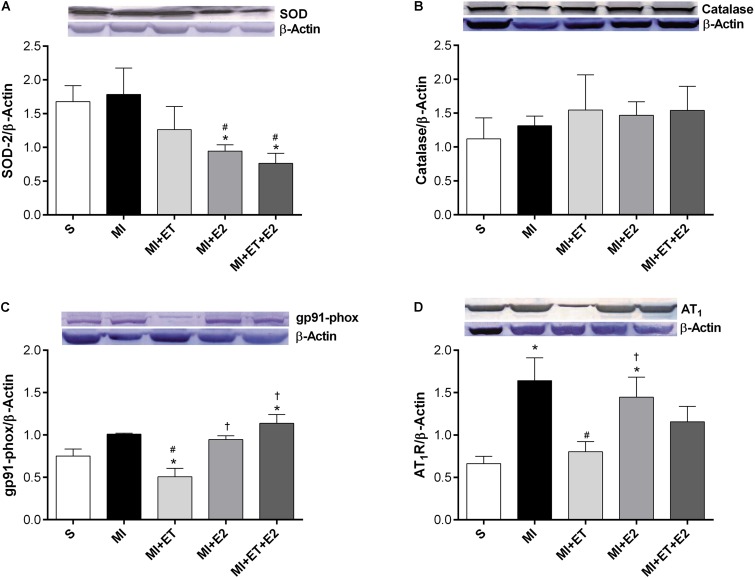
Expression of antioxidant and oxidant proteins. SOD **(A)**, catalase **(B)**, gp91-phox **(C),** and AT-1 receptor **(D)**. Data are expressed as Mean ± SEM. *One-way* ANOVA followed by the Fisher’s *post hoc* test. ^∗^*p* < 0.05 vs. S; ^#^*p* < 0.05 vs. MI; ^†^*p* < 0.05 vs. ET.

Regardless of the treatment, no significant differences in catalase expression between groups were observed, although, a reduction in SOD expression occurred in the groups submitted to E2 treatment (**Figures 6A,B**). However, the infarction induced an increase in the expression of gp91-phox (*p* < 0.05 vs. S), and this response was not prevented in the animals treated with a combination of E2 and ET. Moreover, for treatment with E2 alone, this increase occurred only in comparison with the MI+ET group (*p* < 0.05), with no differences when compared with the remaining groups (**Figure 6C**). As expected, ET reduced gp91-phox expression when compared to the S and MI groups (*p* < 0.05).

The expression of the AT-1 receptor increased after MI (*p* < 0.05 vs. S). ET was able to prevent the increased protein expression of the AT-1 receptor compared with the MI group (*p* < 0.05). However, E2 increased the expression of this receptor (*p* < 0.05 vs. S and MI+ET), but no change in this parameter was observed when combined with ET (**Figure 6D**).

### Antioxidant Enzyme Activity

**FIGURE 7:**
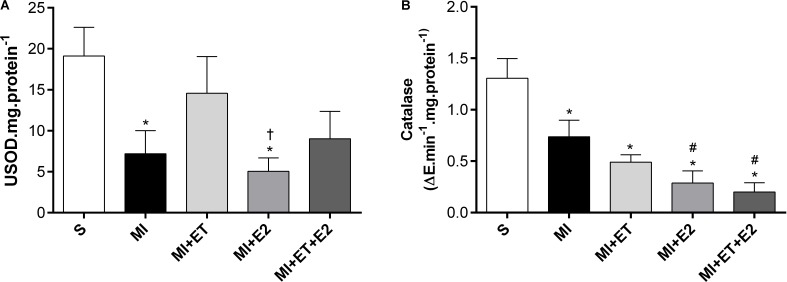
Antioxidant enzymatic activity of SOD **(A)** and Catalase **(B)** in cardiac tissue. Data are expressed as Mean ± SEM. *One-way* ANOVA followed by the Fisher’s *post hoc* test. ^∗^*p* < 0.05 vs. S. ^#^*p* < 0.05 vs. MI. ^†^*p* < 0.05 vs. ET.

Although there was no change in SOD and catalase expression, a reduction in their activity was observed following MI (*p* < 0.05 vs. S) (**Figures 7A,B**). No treatment was capable of reversing the reduction in catalase activity, whereas treatment with E2 did not prevent the reduction in SOD activity compared with the S and ET groups (*p* < 0.05). In addition, ET prevented the SOD activity reduction in the MI+ET and MI+ET+E2 groups.

## Discussion

The main findings of this study are that treatment with E2 does not prevent the worsening of cardiac function in OVX female rats after MI and negatively changes the parameters involved in cardiac remodeling. This result was accompanied by increased collagen deposition and cardiac hypertrophy, which may be explained, at least partially, by an increase in the production of ROS and a concomitant reduction in the activities of antioxidant enzymes, causing increased oxidative stress in the heart. Moreover, the combination of E2 and ET reverses the post-MI functional and morphological changes induced by ET.

Myocardial infarction is the result of an ischemic process that leads to a series of morphofunctional changes, among them increased collagen deposition, heart hypertrophy and changes in LV pressure (Fernandes et al., 2011). The increased collagen deposition plays an important role in the post-MI adverse effects and involves the activation of a cascade of neurohumoral signaling pathways, which lead to the activation of growth mediators in the extracellular matrix (MacKenna et al., 2000). A recent study conducted by Szabó et al. (2018) showed that the reduction in ovarian hormones in OVX female rats increases collagen deposition via a reduction in the activity of matrix metalloproteinase (MMP-2).

The RAAS is one of the pathways involved in the cardiac remodeling process after MI and is implicated in the pathogenesis of diastolic dysfunction (Mehta and Griendling, 2007). Studies show increased RAAS activation and expression of the AT-1 receptor after MI (Sun, 2009) as well as following an ovariectomy (Ricchiuti et al., 2009; Ribeiro-Jr et al., 2012), inducing deleterious cardiac effects. Our results corroborate with previously published data (de Almeida et al., 2014), indicating that the participation of RAAS, via the AT-1 receptor, is associated with post-MI cardiomyocytes hypertrophy and collagen deposition. Moreover, our data demonstrate that post-MI treatment with E2 led to increased AT1 receptor expression. In contrast, a previous study showed that treatment with E2 is capable of regulating or even reducing the AT1 receptor expression in OVX female rats (Dean et al., 2005). However, little is known about the effects of treatment with E2 on the regulation of the expression of this receptor in OVX female rats undergoing MI.

Additionally, a joint system of the activation of oxidative pathways, in particular, related to the gp91-phox enzyme, has already been demonstrated, indicating that the RAAS, via activation of the AT-1 receptor, stimulates oxidative stress through NADPH oxidase (Yung et al., 2011). In the heart, the activation of the AT-1 receptor promotes myocyte hypertrophy through the joint action of the Akt, Rac-1, and gp91-phox pathways (Hingtgen, 2006). In our study, the animals receiving E2 developed an imbalance between the produced ROS. This imbalance may be attributed to the increased expression of the gp91-phox enzyme, which led to increased oxidative stress in the heart post-MI, as shown by increased protein oxidation (AOPP). Altogether, these results may explain the increased collagen deposition and cardiac hypertrophy in the groups receiving treatment with E2. Although the AOPP levels did not change in the group receiving the combination of treatments (MI+ET+E2), it was demonstrated in this study that the increased AT-1 expression receptor in animals after MI occurred together with the increase in gp91-phox expression, indicating the potential regulation of gp91-phox expression induced by AT-1 in these animals. As shown by Fukui et al. (2001), the increased gp91-phox expression is involved in the pathological remodeling of the heart via increased NF-κB activity in the infarction region.

In addition to the cardiac morphometric and molecular changes previously described, the animals treated with E2 presented increased infarction size when compared with the MI and MI+ET groups. Our results corroborate the findings of Smith et al. (2000), who observed increased infarction size and mortality in animals receiving 11 weeks of treatment with E2. Prior data indicate the participation of the AT-1 receptor in the increased infarction size in pigs (Schwarz et al., 1997; Jalowy et al., 1998), confirming the importance of RAAS in the deleterious effects of the therapy since there was an increase in the expression of the AT-1 receptor in the animals treated with E2. These evidences suggest a potential signaling pathway involved in the increase in infarction size in these animals as well as in the worsening of the morphological and functional parameters.

The adverse effects of treatment with E2 after MI on the morphometric and molecular parameters were accompanied by increased LVEDP and Tau values. The LVEDP presents as an important parameter in the evaluation of ventricular function, and in general, its increase is associated with systolic dysfunction and cardiac insufficiency (Pfeffer et al., 1984; Kang et al., 2012; Meric et al., 2014). Diastolic dysfunction refers to mechanical and functional anomalies during LV relaxation and filling (Zhao et al., 2014). Tau is defined as the time constant of isovolumic filling, and its increase is also associated with reduced LV diastolic function (Thomas et al., 1992; Davis et al., 1999; Bai, 2008). Therefore, the results show that E2, alone or in combination with ET, promotes an increase in the LVEDP and Tau, exceeding the values observed in the MI and S groups. Altogether, these results suggest ventricular dysfunction in the animals receiving treatment with E2 regardless of combination with ET.

A previous study conducted by our research group showed that ET is capable of reducing cardiac deleterious functional effects in OVX female rats undergoing MI (de Almeida et al., 2014). However, estrogen therapy for the purposes of the prevention and treatment of CVD is controversial in the literature. Saleh et al. (2000) demonstrated that acute low-dose use of estradiol leads to improvement in the sensitivity of baroreflex in OVX female rats. In addition, when selective estrogen receptors (ER) agonists and oxytocin are employed in conjunction with swimming training in OVX rats for 4 weeks before the induction of an ischemia and reperfusion protocol, Bulut et al. (2016) found improvements in cardiac function and in proinflammatory markers. These results suggest that the activation of ER would be beneficial to protect against myocardial ischemia in post-menopausal women. Other study pointed out to the important role of ER-β in mediates the beneficial effects of E2 since its deletion induce an aggravation of heart failure markers and increase mortality in mice knockout for ER-β during E2 treatment (Pelzer et al., 2005). However, other studies indicate deleterious cardiovascular effects of estrogen therapy in ovariectomized rats (Van Eickels et al., 2003; Ricchiuti et al., 2009), suggesting that E2 may increase the incidence of CVD in women.

Another fact that should be taken into account is related to the dose-dependent effect of E2 therapy. A study performed by Meng et al. (2011) in ovariectomized mice verified some important alterations in the kidneys of animals treated with moderate and high doses of E2, although, they did not find any effects on the cardiac function of these animals. In MI ovariectomized mice, Zhan et al. (2008) evaluated the effects of treatment with low, moderate and high doses of E2 for 8 weeks before the MI induction and observed that the moderate and high doses exacerbate cardiac fibrosis, hypertrophy, dysfunction and dilatation associated with damages in other organs. These results demonstrate that estrogen therapy with dosages that raise the plasma E2 concentration far beyond the physiological levels can be detrimental to the heart. However, Van Eickels et al. (2003), using a low dosage of E2 (4 μg/d), which restore the physiological concentration, demonstrated an increase in the LV remodeling and in mortality post-MI. Therefore, the cardiovascular effects of estrogen therapy are quite complex, which increases in complexity when evaluated after MI and in association with other therapies, such as ET.

### Limitations

A potential limitation of our study is that we did not evaluate the cardiac effects of E2 therapy with different doses. However, we used an estrogen therapy protocol that is capable to maintain the physiological plasmatic concentration of E2 observed in control female rats not subjected to ovariectomy, as showed previously (Saengsirisuwan et al., 2009; Claudio et al., 2013; Endlich et al., 2017).

### Future Directions

Although we have demonstrated important effects of E2 therapy on cardiac function and remodeling after MI, some questions remain to be elucidated. One of them is the question of dose-dependency of E2 effects, to evaluate if different concentrations would elicit the same cardiac effects as observed in this study and what was the interaction between different dosages of E2 with ET. In addition, it would be important to investigate the cardiac effects of different preparations of hormone therapy after MI, including progestins, in this model of estrogen deficiency.

## Conclusion

We conclude that the use of E2 therapy is not able to prevent or reverse the MI-induced cardiac changes in diastolic dysfunction and worsens the parameters related to cardiac remodeling. Moreover, when combined with ET, E2 impairs the positive changes promoted by ET in female OVX MI rats.

## Author Contributions

SdA contributed to the design of the study, data acquisition, data analysis, and manuscript writing. EC contributed to the design of the study, data analysis, and manuscript writing. VM contributed to western blot analysis and manuscript writing. GB contributed to data analysis and manuscript writing. EM contributed to morphological and data analysis. PP contributed to morphological and data analysis. JG contributed to study design and manuscript writing. SG contributed to data analysis and manuscript writing. GdA contributed to study design, financial support, data analysis, and manuscript writing.

## Conflict of Interest Statement

The authors declare that the research was conducted in the absence of any commercial or financial relationships that could be construed as a potential conflict of interest.
